# Diagnosis of foreign body aspiration with ultralow-dose CT using a tin filter: a comparison study

**DOI:** 10.1007/s10140-020-01764-7

**Published:** 2020-03-09

**Authors:** Lena Gordon, Patrik Nowik, Shahla Mobini Kesheh, Marika Lidegran, Sandra Diaz

**Affiliations:** 1 Department of Pediatric Radiology, Astrid Lindgren Children’s Hospital, Karolinska University Hospital, Stockholm, Sweden; 2 Medical Radiation Physics and Nuclear Medicine, Karolinska University Hospital, Stockholm, Sweden; 3 Department of Clinical Science, Intervention and Technology, Karolinska Institutet, Stockholm, Sweden; 4 Department of Diagnostic Radiology, Skane University Hospital, Malmo, Sweden

**Keywords:** Ultralow-dose CT, Foreign body aspiration, Bronchoscopy, Fluoroscopy, Chest radiography

## Abstract

**Purpose:**

Suspected airway foreign body aspiration (FBA) is a common event in paediatric emergency units, especially in children under 3 years of age. It can be a life-threatening event if not diagnosed promptly and accurately. The purpose of this study is to compare the diagnostic performance of an ultralow-dose CT (DLP of around 1 mGycm) with that of conventional radiographic methods (fluoroscopy and chest radiography of the airways) in the diagnosis of FBA children’s airways.

**Methods:**

Retrospective cross-sectional study. Data from 136 children were collected: 75 were examined with conventional radiographic methods and 61 with ultralow-dose CT. Effective doses were compared using independent *t* tests. The results of bronchoscopy, if performed, were used in creating contingency 2 × 2 tables to assess the diagnostic performance between modalities. An extra triple reading of all images was applied for this purpose.

**Results:**

The effective doses used in the ultralow-dose CT examinations were lower compared with those in conventional methods (*p* < 0.001). The median dose for CT was 0.04 mSv compared with 0.1 mSv for conventional methods. Sensitivity and specificity were higher for ultralow-dose CT than those for conventional methods (100% and 98% versus 33% and 96%) as were the positive and negative predicted values (90% and 100% versus 60% and 91%).

**Conclusion:**

Ultralow-dose CT can be used as the imaging of choice in the diagnosis of airway FBA in emergency settings, thereby avoiding concerns about radiation doses and negative bronchoscopy outcomes.

## Introduction

Foreign body aspiration (FBA) is a common paediatric emergency. According to the Centre for Disease Control in the USA, it is the fourth most common cause of death in children between 1 and 5 years of age [1]. Tracheobronchial FBA commonly occurs in children younger than 3 years of age. The degree of severity depends on the location where the FB is deposited in the airway and the size, shape and type of material aspirated. The symptoms and management vary depending on whether or not the event has been observed by the parents or caregivers. The diagnosis of airway FBA can be challenging in both situations. However, an early diagnosis is essential to avoid dramatic consequences such as death or irreversible lung/airway damage and to prevent complications.

Radiography of the airways is usually the first-line imaging modality when airway FBA is suspected. It is inexpensive and widely available. Many centres perform chest radiographs in full inspiration and forced expiration and some include lateral decubitus views. In other hospitals, chest radiographs are followed by airway fluoroscopy which allows visualization of displacement of the mediastinum and diaphragm. The use of conventional radiographic methods to diagnose airway FBA requires skill and experience. Fluoroscopy is an operator-dependent examination that requires expertise. It can be difficult for an unexperienced on-call resident to detect subtle movements of the mediastinum and diaphragm in an irritated and uncooperative child. Radiography can detect air trapping, atelectasis, consolidations, hyperaeration and segmental or lobar collapse, but all these findings are non-specific for FBA. Furthermore, approximately 90% of aspirated foreign bodies are radiolucent, such as food and plastic particles, and consequently not visible with conventional methods. All these reasons make the diagnosis of FBA challenging; therefore, the correct treatment might be delayed.

Bronchoscopy, flexible or rigid, is considered the standard technique for extraction of bronchial foreign bodies in children. However, this invasive technique requires general anaesthesia and, as with all interventional procedures, is subject to risk for complications. Its use should therefore be restricted to treatment when airway FBA has been correctly diagnosed.

Computed tomography (CT) with multiplanar reconstruction has been incorporated into the diagnosis of airway FBA in the last decade. Rapid technological advancement has allowed the development of multidetector CT with a significant reduction in the acquisition time and a marked improvement in the quality of the images, while lowering the radiation doses [2]. CT has mainly been used to identify patients who require bronchoscopy.

The use of CT as the first line in the diagnosis of airway FBA has probably not been extended due to the high radiation dose attributed to this modality. Recently, CT examinations of the chest have been possible using very low radiation doses (0.06 mSv) using a tin-filtrated X-ray beam [3]. A tin filter shapes the X-ray spectrum, filtering mostly low-energy photons, which leaves a narrow, high-energy spectrum. It is optimized for imaging of high-contrast tissues (such as the airways). Beam hardening is reduced thanks to the shape of the tin-filtered spectrum, reducing artefacts from bone.

The purpose of this study was to compare the diagnostic performance of an ultralow-dose CT protocol (DLP of around 1 mGycm) dose equivalent to conventional radiographic methods (fluoroscopy and chest radiography) in the diagnosis of FBA of the airways in children.

## Materials and methods

The study was approved by the regional ethics committee. This is a retrospective study, from January 2014 to December 2018. All patients were referred from the paediatric emergency department with a witnessed or suspicion of FBA. The patients were identified by searching all fluoroscopy, chest radiography and CT reports using the phrase “foreign body” and in order to not miss any patients, billing codes were also used. The data collected were age, sex, standard chest radiography data, standard fluoroscopy data, CT data and endoscopy results if performed. At our department, we carry out *double reading* of all cases regardless of whether they have been read by a resident or consultant. For the purpose of this study, a third reading was made by a senior consultant with 25 years of experience in paediatric radiology. Before the use of ultralow-dose CT, the routine at our institution was chest radiography in combination with fluoroscopy.

### Chest radiography and fluoroscopy

We identified 75 children examined between January 2014 and December 2016 with chest radiography of the upper and lower airways and fluoroscopy of the chest. A dedicated paediatric chest X-ray equipment (Digital Diagnost 4.1, Philips Healthcare, Eindhoven, The Netherlands) was used for radiography procedures and a dedicated paediatric fluoroscopy equipment (Artis Zee, Siemens Healthcare, Forchheim, Germany) was used for fluoroscopy procedures.

The examination protocol used for the chest radiography was adapted for the child’s age with automatic exposure designed to deliver the optimal image quality irrespective of patient size where the automatic exposure control (AEC) regulates the milliampere-second (mAs). The tube voltage used for fluoroscopy and radiography examination was between 75 and 80 kV. The beam filtrations ranged from 4 mm Al for radiography up to 4 mm Al + 0.1 mm Cu for fluoroscopy procedures.

The examination protocol for the fluoroscopy procedures was a lung fluoroscopy program designed with 15 pulses per second. The reason for the high pulse rate is to catch the flow of the respiratory function, which is faster in children compared with adults. Radiography systems have age-dependent examination protocols that are appropriate to the patient and give a baseline value for the current patient group. Furthermore, the system has an advanced AEC which regulates the dose individually for every patient by changing different parameters based on patient thickness.

Information from the radiation dose structured reports (RDSRs) were used to estimate the effective doses using a commercial Monte Carlo (MC) simulation package (PCXMC 2.0, STUK, Helsinki, Finland) [4].

### Ultralow-dose CT of the chest

We identified 61 children examined between January 2017 and December 2018 with ultralow-dose CT of the airways. A foreign body ultralow-dose CT scan protocol was set up on a dedicated paediatric CT scanner (SOMATOM Force, Siemens Healthcare, Forchheim, Germany), using the following scan protocol parameters: 100 kV with tin filtration, 57.6 mm collimation, a dual-source protocol with a pitch of 3.2, 0.25 s rotation time and automated tube current modulation (organ characteristic: thorax and a reference mAs of 30, with reference mAs being the vendor’s image quality reference parameter). *Z*-axis coverage is from the larynx to the base of the lungs.

The ultralow-dose CT scan was performed without injection of intravenous contrast and without sedation or anaesthesia. Non-cooperative children were held with help from their parents or a radiographer in the same manner as when taking a plain radiograph.

The effective dose to each patient from the CT scans was estimated using the commercial software package ImpactDose (Advanced Breast-CT GmbH, Erlangen, Germany), which uses pre-calculated MC conversion factors.

### Bronchoscopy

Direct bronchoscopy was performed in patients with radiological signs of FBA or patients with negative radiology but symptoms remaining. All bronchoscopies were performed under general anaesthesia by otolaryngologists according to local routines. Patients with normal radiological findings and without any remaining clinical symptoms were sent home with the information to return to the hospital in case of recurring respiratory symptoms.

Results from bronchoscopy and follow-up at the emergency unit were used as determinants of the diagnostic performance in both the group of children who underwent fluoroscopy and chest radiography and the group who underwent ultralow-dose CT of the airways. True positive results were those interpreted as suggesting a foreign body in the airway by fluoroscopy and chest radiography of the chest or by ultralow-dose CT of the airways that was confirmed by bronchoscopy. False positive results were those interpreted as suggesting a foreign body in the airway by fluoroscopy and chest radiography or by ultralow-dose CT of the airways that could not be confirmed by bronchoscopy. True negative results were those interpreted as negative by fluoroscopy and chest radiography or ultralow-dose CT of the airways and corroborated by bronchoscopy or by clinical follow-up at the emergency unit. False negative results were those interpreted as negative by fluoroscopy and chest radiography or by ultralow-dose CT of the airways but refuted by the observation of foreign body under clinical follow-up or bronchoscopy.

Statistical analysis was performed using SPSS version 25.0 for Windows (IBM Corp., Armonk, NY, USA). Sensitivity, specificity, and positive and negative predictive values for both the fluoroscopy/radiography group and the ultralow-dose CT group were calculated from a 2 × 2 contingency table. The *t* test for independent samples was used to calculate statistical difference of effective doses between groups.

## Results

A total of 136 children were included in the study. Mean age was 2 years and 2 months ± 1.6 months with a minimum age of 2 months and maximum age of 7 years and 7 months. More than 90% of the children were under 4 years old. The male to female ratio was 1:5.

The mean incident dose received in the group of 75 children who underwent chest radiography and fluoroscopy was 0.13 ± 0.03 mGy (range 0.1 to 0.16 mGy) and the mean effective dose was 0.12 ± 0.56 mSv (95% confidence interval for the mean, 0.11 to 0.13 mSv) with median of 0.10 mSv.

The mean CTDI_VOL_ (32 cm) and DLP (32 cm) in the group of 62 children who underwent ultralow-dose CT of the airways were 0.03 ± 0.009 mGy (range 0.02 to 0.06 mGy) and 0.99 ± 0.31 mGy (range 0.45 to 1.93 mGy) respectively and the mean effective dose was 0.04 ± 0.12 mSv (95% confidence interval for the mean, 0.03 to 0.04 mSv) with median 0.04 mSv.

The diagnostic performance of fluoroscopy combined with chest radiography of the airway and ultralow-dose CT of the airways is shown in Table 1. Sensitivity, specificity, and predictive positive and negative values as well as accuracy were higher in the ultralow-dose CT of the airways group.

**Table 1:** Diagnostic characteristics of fluoroscopy combined with chest radiography and low-dose CT of the airways for the detection of foreign body. *CI*, confidence interval (%)

Diagnostic performance	Fluoroscopy and radiography of the chest	Very low-dose CT of the chest
	Value% (95% CI)	Value% (95% CI)
Sensitivity	33 (7 to 70)	100 (66 to 100)
Specificity	96 (89 to 99)	98 (90 to 99)
Positive predicted value	60 (22 to 88)	90 (56 to 98)
Negative predicted value	91 (87 to 94)	100
Accuracy	89 (80 to 95)	98 (91 to 99)

The percent of misdiagnosed cases in the fluoroscopy and chest radiography group was 11% compared with 3% in the ultralow-dose CT airways group. Subtle mediastinal shift was the most often misdiagnosed sign of FBA by fluoroscopy (6 cases). Concerning chest radiography, non-radiopaque FBA and absence of air trapping were commonly reported. Few cases with atelectasis and consolidations were reported as foreign body suspects.

**Fig. 1:**
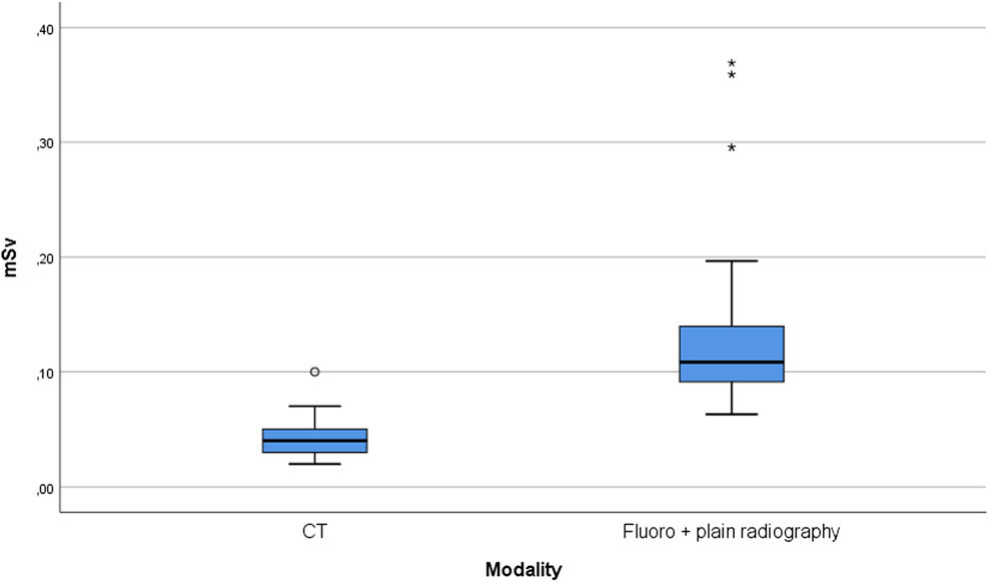
Box plot of total mSv for ultralow-dose CT and combined fluoroscopy and chest radiography

There was a statistically significant difference in the effective dose used by ultralow-dose CT of the airways related to fluoroscopy and chest radiography (*p* < 0.001) (Fig. 1).

According to the medical journals, none of those patients who went home without radiological or clinical symptoms returned to the emergency unit.

**Fig. 2:**
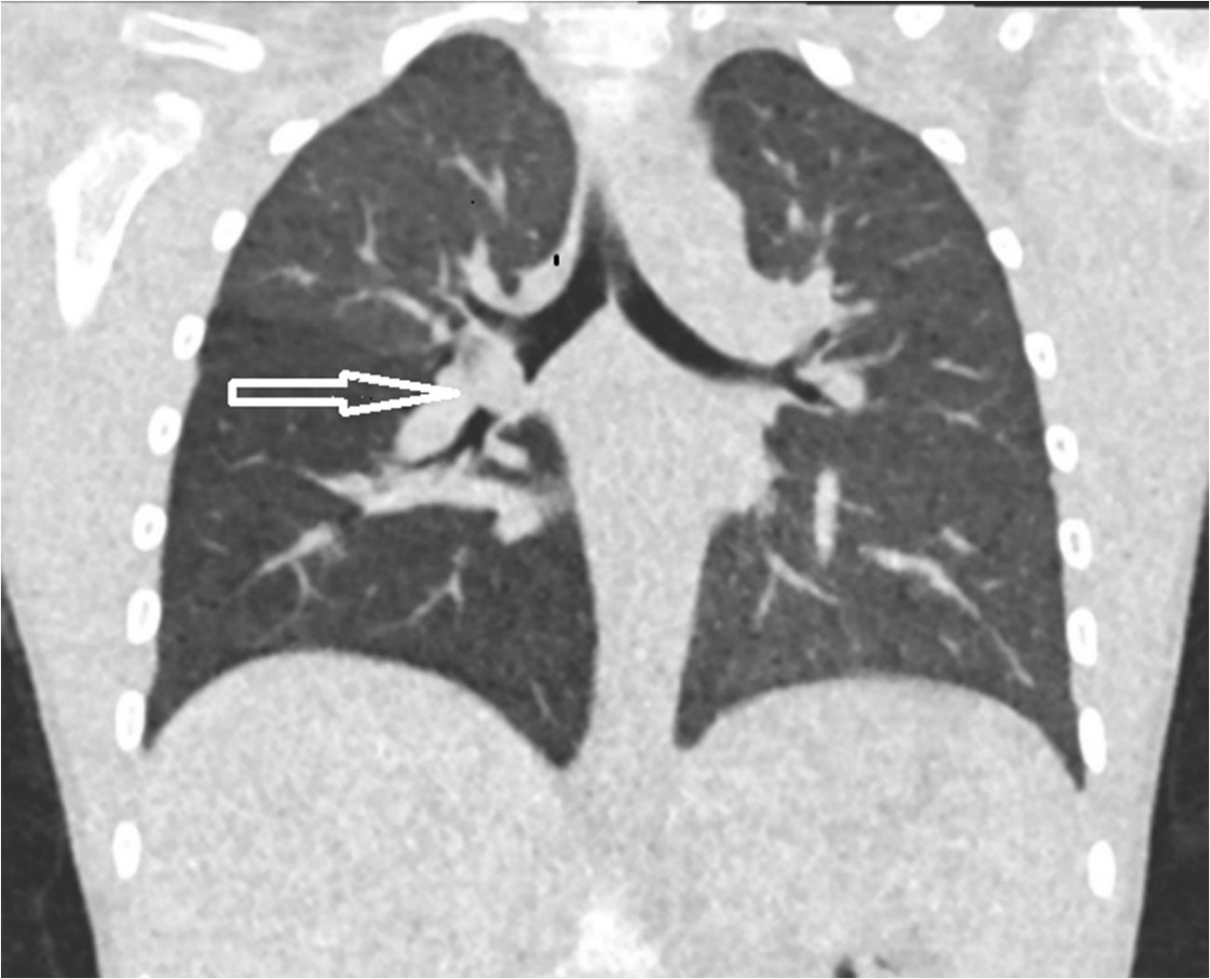
7-year-old child with a piece of carrot in the right main bronchus (arrow). Effective dose: 0.04 mSv

**Fig. 3:**
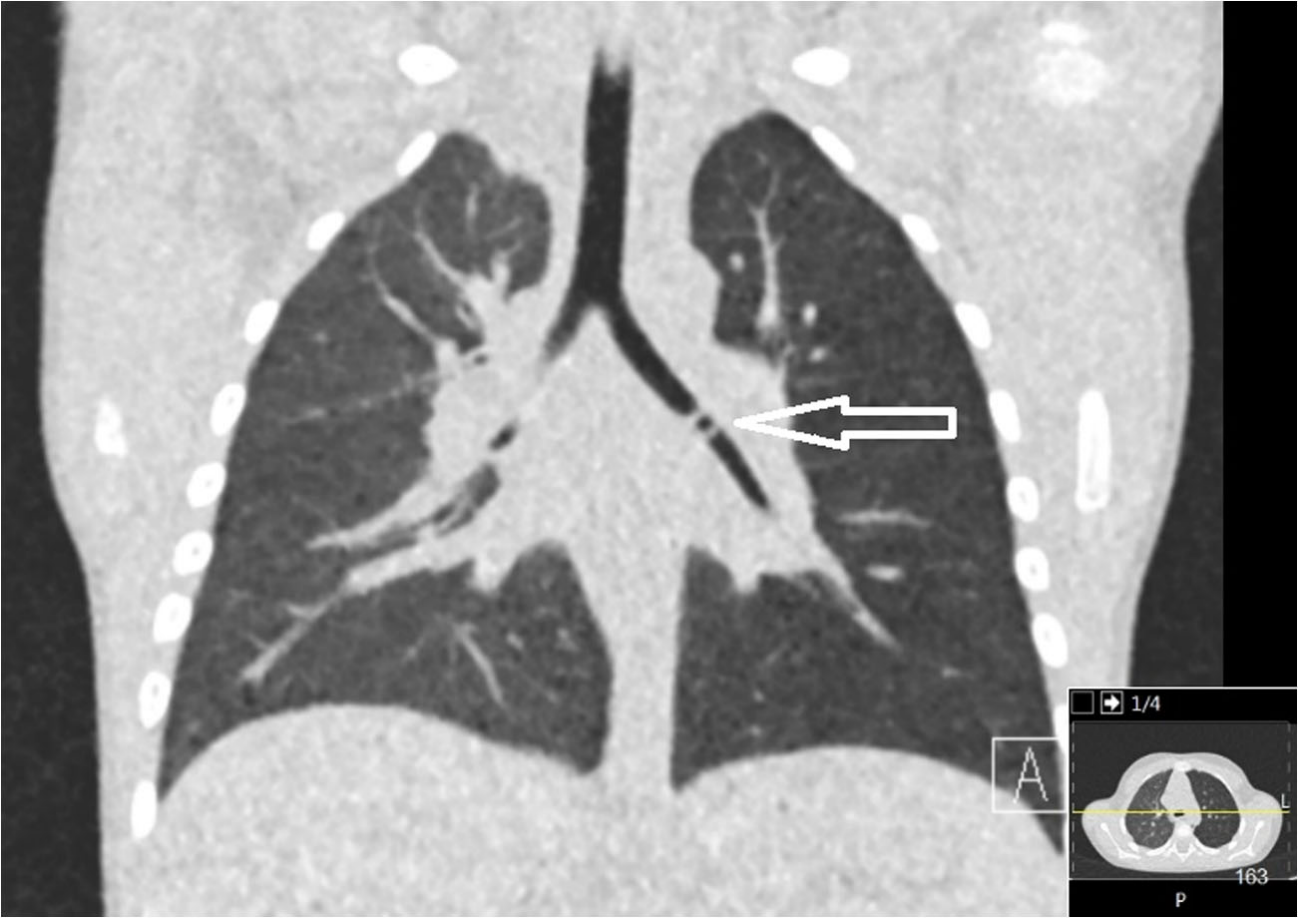
2-year-old child with a Perler bead in the left main bronchus (arrow). Effective dose: 0.04 mSv

A total of 10 bronchoscopies were performed in the fluoroscopy/chest radiography group (2 false positives and 5 false negatives) and 10 in the ultralow-dose CT group (1 false positive and no false negative). The most common findings were food particles, for example nuts, seeds or pieces of carrot (Fig. 2) followed by small objects like Lego® or Perler beads (Fig. 3).

## Discussion

Aspiration of a foreign body in the airways is a critical and life-threatening situation that requires prompt diagnosis and correct management to avoid lethal consequences. Plain chest radiography has been the most common first-line modality in the diagnosis of FBA, sometimes in combination with fluoroscopy to improve accuracy. Previous studies have proved CT to be highly accurate in the diagnosis of FBA in children. However, the use of CT as the first-line method in the diagnosis of airway FBA has until now not been widespread, probably due to the high radiation dose attributed to this modality.

Review of the last 15 years of literature revealed several papers related to the use of CT in the diagnosis of FBA [2, 5–14]. The focus has generally been on the reduction of false positives or negatives compared with conventional techniques to minimize the number of bronchoscopies performed. There are fewer publications regarding the use of low-dose CT in the diagnosis of FBA [15–18].

In our study, we used a tailored ultralow-dose protocol with a tin filter to reduce radiation dose, aiming for a dose equivalent to that of a plain chest radiograph. The achieved dose proved to be significantly lower for CT, using this protocol, compared with conventional radiographic methods combining fluoroscopy and chest radiography.

Our study showed the same high sensitivity and specificity (100% versus 98%) as other studies [13, 18] with a mean effective dose nearly a thirtieth of the dose described in a recent published paper on the subject [19].

**Fig. 4:**
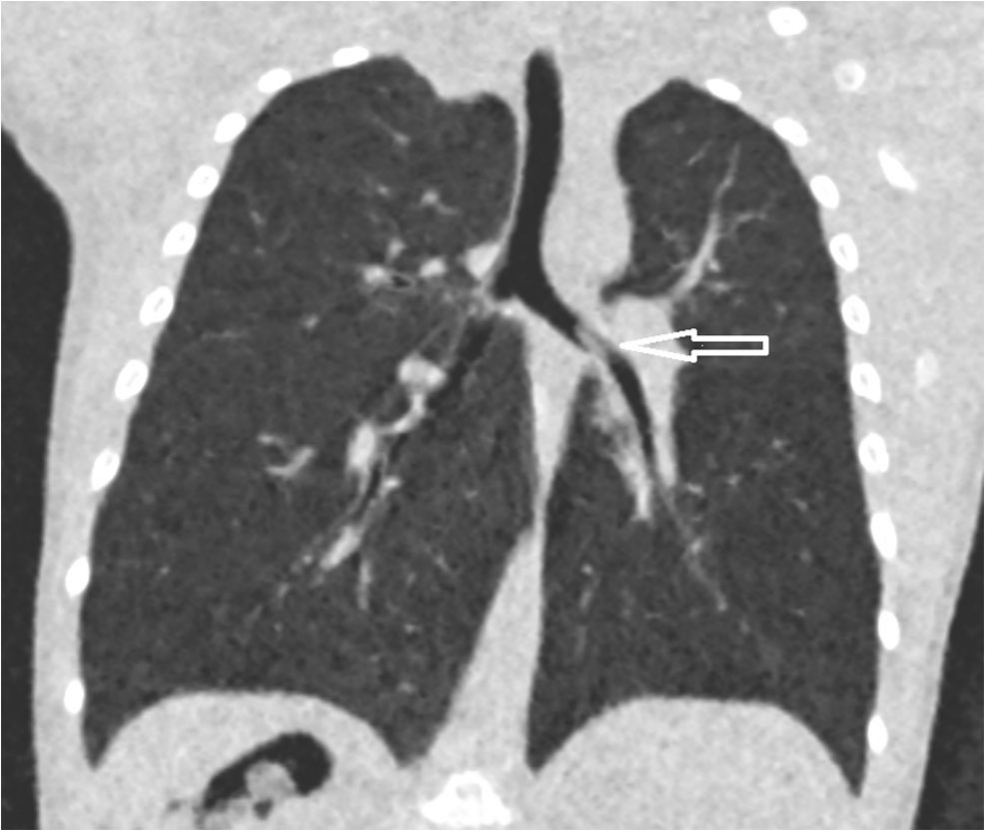
1-year-old child with a stearin ball in the left main bronchus (arrow). Effective dose: 0.06 mSv

Only one case was regarded as false positive but questionably so. It occurred in a child with a history of candle-grease aspiration. At the time the ultralow-dose CT was performed, a stearin ball could be visualized in the left main bronchus (Fig. 4). The bronchoscopy was delayed more than 12 h due to overload of work for the anaesthesia team and the stable condition of the child. After this delay, the bronchoscopy turned out to be negative. Our theory is that the child coughed up the small piece of stearin while waiting.

Previous studies have shown that CT of the airways is highly sensitive in the diagnosis of FBO due to the high contrast between air and any particle with a higher attenuation. In our study, no foreign bodies were missed. However, vegetable fragments, missed by CT, have been reported in the literature [6]. We consider it unlikely that FBA in the airways such as plastic pieces may be missed using CT.

Our results show that an ultralow-dose CT protocol can reduce the effective radiation dose to a level equivalent to that of an anteroposterior and lateral standard chest radiography alone, maintaining the high diagnostic accuracy. In addition, other findings such as consolidations, atelectasis and pleural effusion were reported.

At our hospital, the routine is to do bronchoscopy when the patient has positive radiological findings and/or persistent symptoms.

The major limitation of our study is the retrospective nature. Any deviations of the clinical protocol were impossible to detect. Furthermore, only a minority of patients underwent bronchoscopy: patients diagnosed as positive of FBA or patients diagnosed as negative but with a strong clinical suspicion. Thus, some false negative cases could have been missed on clinical follow-up. We have one false positive case using our ultralow-dose CT protocol (stearin ball). However, we consider it a dubious case caused by a delay between CT and bronchoscopy. Finally, the CT dose estimation software package used in this study does not take the patient table into account, which can lead to an overestimation of the effective doses of around 5% [20].

## Conclusion

Ultralow-dose CT of the airways using a tin filter with a dose equivalent to conventional radiographic methods reduces radiation dose significantly compared with previously described low-dose CT protocols, maintaining the high sensitivity and specificity for detection of aspirated foreign bodies. It can be used as a first and only diagnostic tool in emergency settings, decreasing the risk of misdiagnoses and negative bronchoscopy outcomes and thereby avoiding operative risks and costs.
